# Diaqua­(nitrato-κ^2^ *O*,*O*′)[2-(1*H*-1,2,4-triazol-1-yl-κ*N* ^2^)-1,10-phenanthroline-κ^2^ *N*,*N*′]cadmium(II) nitrate

**DOI:** 10.1107/S1600536810039735

**Published:** 2010-10-13

**Authors:** Shi Guo Zhang, Hui Ming Zhang

**Affiliations:** a Binzhou Key Laboratory of Material Chemistry, Department of Chemistry and Chemical Engineering, Binzhou University, Binzhou 256603, People’s Republic of China

## Abstract

In the title complex, [Cd(C_14_H_9_N_5_)(NO_3_)(H_2_O)_2_]NO_3_, the Cd^II^ ion is coordinated in a distorted penta­gonal-bipyramidal geometry. The equatorial sites are occupied by a 2-(1*H*-1,2,4-triazol-1-yl)-1,10-phenanthroline ligand in a tridentate coordination mode and a bis-chelating nitrate ligand. Two aqua ligands are coordinated at the axial sites. All non-H atoms in the equatorial plane are co-planar within 0.0673 Å. In the crystal, inter­molecular O—H⋯O and O—H⋯N hydrogen bonds connect the components into a two-dimensional network parallel to (001). In addition, there is a π–π stacking inter­action between symmetry-related benzene rings, with a centroid–centroid distance of 3.598 (3) Å.

## Related literature

For related structures, see: Li (2009 ▶); Xie *et al.* (2009 ▶).
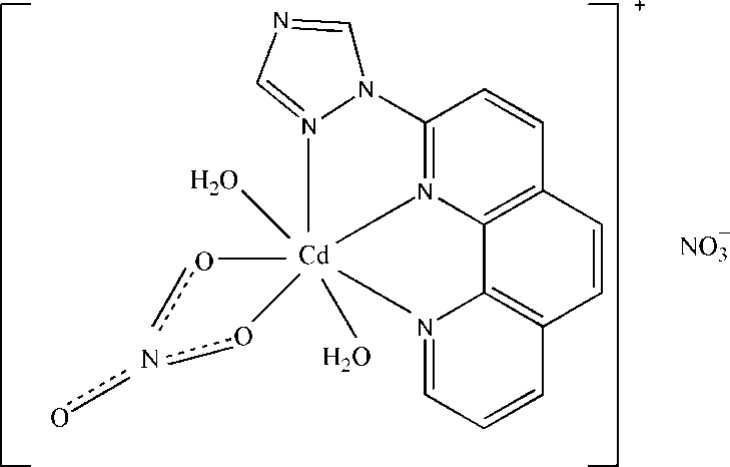


## Experimental

### 

#### Crystal data

[Cd(C_14_H_9_N_5_)(NO_3_)(H_2_O)_2_]NO_3_
*M* *_r_* = 519.71
Triclinic, 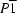

*a* = 8.9934 (18) Å
*b* = 9.1995 (19) Å
*c* = 11.460 (2) Å
α = 88.334 (3)°
β = 72.946 (3)°
γ = 83.375 (3)°
*V* = 900.4 (3) Å^3^
*Z* = 2
Mo *K*α radiation
μ = 1.28 mm^−1^
*T* = 298 K
0.25 × 0.10 × 0.08 mm

#### Data collection

Bruker SMART APEX CCD diffractometer
Absorption correction: multi-scan (*SADABS*; Sheldrick, 1996 ▶) *T*_min_ = 0.741, *T*_max_ = 0.905
4496 measured reflections
3311 independent reflections
2954 reflections with *I* > 2σ(*I*)
*R*_int_ = 0.020

#### Refinement

*R*[*F* ^2^ > 2σ(*F* ^2^)] = 0.044
*wR*(*F* ^2^) = 0.104
*S* = 1.07
3311 reflections
271 parameters
H-atom parameters constrained
Δρ_max_ = 0.78 e Å^−3^
Δρ_min_ = −0.56 e Å^−3^

### 

Data collection: *SMART* (Bruker, 1997 ▶); cell refinement: *SAINT* (Bruker, 1997 ▶); data reduction: *SAINT*; program(s) used to solve structure: *SHELXTL* (Sheldrick, 2008 ▶); program(s) used to refine structure: *SHELXTL*; molecular graphics: *SHELXTL*; software used to prepare material for publication: *SHELXTL*.

## Supplementary Material

Crystal structure: contains datablocks I, global. DOI: 10.1107/S1600536810039735/lh5140sup1.cif

Structure factors: contains datablocks I. DOI: 10.1107/S1600536810039735/lh5140Isup2.hkl

Additional supplementary materials:  crystallographic information; 3D view; checkCIF report

## Figures and Tables

**Table 1:** Hydrogen-bond geometry (Å, °)

*D*—H⋯*A*	*D*—H	H⋯*A*	*D*⋯*A*	*D*—H⋯*A*
O8—H5⋯O5^i^	0.75	2.05	2.752 (6)	156
O8—H4⋯O2^ii^	0.86	2.03	2.865 (5)	163
O7—H7⋯O3^iii^	0.78	2.59	3.269 (6)	147
O7—H7⋯O1^iii^	0.78	2.22	2.966 (5)	160
O7—H6⋯N5^iv^	0.99	2.47	3.420 (6)	162
O7—H6⋯O6^iv^	0.99	2.45	3.227 (6)	135
O7—H6⋯O4^iv^	0.99	1.83	2.802 (6)	167

Symmetry codes: (i) 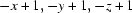; (ii) 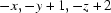; (iii) 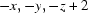; (iv) 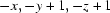.

**Fig. 1.**
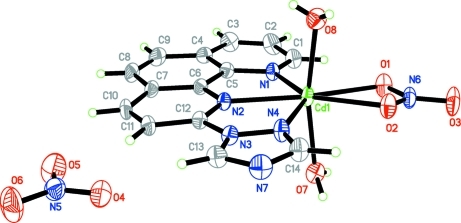
The asymmetric unit of the title compound with displacement ellipsoids shown at the 30% probability level

## supplementary crystallographic information

### Comment

Derivatives of 1,10-phenanthroline play an important role in modern coordination chemistry and to our knowledge only two complexes with 2-(1*H*-1,2,4-triazol-1-yl)-1,10-phenanthroline as ligand have been published up to date (Li, 2009; Xie *et al.* 2009). Our interest in the correlation between the coordination geometry and the counter ion resulted in us synthesizing the title complex and herein we report its crystal structure.

The molecular structure of the title compound is shown in Fig. 1. The Cd^II^ ion is in a distorted pentagonal bipyramidal coordination environment, with two H_2_O ligands in the axial positions. Except for the two water molecules, all non-hydrogen atoms of the cation define a plane within 0.0673 Å with a maximum deviation of -0.1532 (39) Å for atom N5. In the crystal structure, hydrogen bonds involving coordinated water molecules, nitrato ligands and nitrate anions connect the components of the structure into a two-dimensional network parallel to (001). In addition, there is a π–π stacking interaction involving symmetry-related complexes, the relevant distances being *Cg*1···*Cg*1^i^ = 3.598 (3) Å and *Cg*1···*Cg*1^i^_perp_ = 3.428 Å [symmetry code: (i) 1 - *x*, -*y*, 1 - *z*; *Cg*1 is the centroid of C4—C9 ring].

### Experimental

A 5 ml water solution of Cd(NO_3_)_2_4H_2_O (0.0742 g, 0.240 mmol) was added into a 15 ml methanol solution containing 2-(1*H*-1,2,4-triazol-1-yl)-1,10-phenanthroline (0.0598 g, 0.242 mmol) and the mixture was stirred for a few minutes. Colorless single crystals were obtained after the filtrate had been allowed to stand at room temperature for two weeks.

### Refinement

The water H atoms were located in a difference Fourier map and refined as riding in their 'as found' positions with *U*_iso_ = 1.5*U*_eq_(O). Other H atoms were placed in calculated positions and refined as riding with C—H = 0.93 Å, *U*_iso_ = 1.2*U*_eq_(C).

### Crystal data

**Table:**

[Cd(C_14_H_9_N_5_)(NO_3_)(H_2_O)_2_]NO_3_	*Z* = 2
*M**_r_* = 519.71	*F*(000) = 516
Triclinic, *P*1	*D*_x_ = 1.917 Mg m^−^^3^
Hall symbol: -P 1	Mo *K*α radiation, λ = 0.71073 Å
*a* = 8.9934 (18) Å	Cell parameters from 1655 reflections
*b* = 9.1995 (19) Å	θ = 2.6–24.8°
*c* = 11.460 (2) Å	µ = 1.28 mm^−^^1^
α = 88.334 (3)°	*T* = 298 K
β = 72.946 (3)°	Block, colorless
γ = 83.375 (3)°	0.25 × 0.10 × 0.08 mm
*V* = 900.4 (3) Å^3^	

### Data collection

**Table:**

Bruker SMART APEX CCD diffractometer	3311 independent reflections
Radiation source: fine-focus sealed tube	2954 reflections with *I* > 2σ(*I*)
graphite	*R*_int_ = 0.020
φ and ω scans	θ_max_ = 25.8°, θ_min_ = 1.9°
Absorption correction: multi-scan (*SADABS*; Sheldrick, 1996)	*h* = −10→10
*T*_min_ = 0.741, *T*_max_ = 0.905	*k* = −9→11
4496 measured reflections	*l* = −14→12

### Refinement

**Table:**

Refinement on *F*^2^	Primary atom site location: structure-invariant direct methods
Least-squares matrix: full	Secondary atom site location: difference Fourier map
*R*[*F*^2^ > 2σ(*F*^2^)] = 0.044	Hydrogen site location: inferred from neighbouring sites
*wR*(*F*^2^) = 0.104	H-atom parameters constrained
*S* = 1.07	*w* = 1/[σ^2^(*F*_o_^2^) + (0.0541*P*)^2^ + 0.1075*P*] where *P* = (*F*_o_^2^ + 2*F*_c_^2^)/3
3311 reflections	(Δ/σ)_max_ < 0.001
271 parameters	Δρ_max_ = 0.78 e Å^−^^3^
0 restraints	Δρ_min_ = −0.56 e Å^−^^3^

### Special details

**Table:**

Geometry. All e.s.d.'s (except the e.s.d. in the dihedral angle between two l.s. planes) are estimated using the full covariance matrix. The cell e.s.d.'s are taken into account individually in the estimation of e.s.d.'s in distances, angles and torsion angles; correlations between e.s.d.'s in cell parameters are only used when they are defined by crystal symmetry. An approximate (isotropic) treatment of cell e.s.d.'s is used for estimating e.s.d.'s involving l.s. planes.
Refinement. Refinement of *F*^2^ against ALL reflections. The weighted *R*-factor *wR* and goodness of fit *S* are based on *F*^2^, conventional *R*-factors *R* are based on *F*, with *F* set to zero for negative *F*^2^. The threshold expression of *F*^2^ > σ(*F*^2^) is used only for calculating *R*-factors(gt) *etc*. and is not relevant to the choice of reflections for refinement. *R*-factors based on *F*^2^ are statistically about twice as large as those based on *F*, and *R*- factors based on ALL data will be even larger.

### Fractional atomic coordinates and isotropic or equivalent isotropic displacement parameters (Å^2^)

**Table:**

	*x*	*y*	*z*	*U*_iso_*/*U*_eq_	
C1	0.4138 (6)	−0.0355 (5)	0.8867 (5)	0.0444 (12)	
H1	0.3374	−0.0416	0.9611	0.053*	
C2	0.5485 (7)	−0.1354 (6)	0.8625 (5)	0.0556 (14)	
H2	0.5602	−0.2068	0.9192	0.067*	
C3	0.6599 (6)	−0.1269 (6)	0.7571 (5)	0.0528 (14)	
H3	0.7517	−0.1907	0.7414	0.063*	
C4	0.6401 (5)	−0.0228 (5)	0.6697 (4)	0.0400 (11)	
C5	0.5015 (5)	0.0732 (5)	0.7005 (4)	0.0337 (10)	
C6	0.4752 (5)	0.1791 (5)	0.6135 (4)	0.0325 (10)	
C7	0.5814 (5)	0.1851 (5)	0.4961 (4)	0.0365 (10)	
C8	0.7209 (5)	0.0835 (6)	0.4682 (5)	0.0463 (13)	
H8	0.7933	0.0848	0.3914	0.056*	
C9	0.7484 (6)	−0.0130 (6)	0.5511 (5)	0.0506 (14)	
H9	0.8411	−0.0758	0.5308	0.061*	
C10	0.5412 (6)	0.2866 (6)	0.4149 (4)	0.0432 (12)	
H10	0.6071	0.2899	0.3356	0.052*	
C11	0.4082 (6)	0.3809 (5)	0.4486 (4)	0.0404 (11)	
H11	0.3822	0.4497	0.3946	0.048*	
C12	0.3123 (5)	0.3697 (5)	0.5679 (4)	0.0333 (10)	
C13	0.1087 (6)	0.5780 (5)	0.5609 (5)	0.0439 (12)	
H13	0.1516	0.6105	0.4818	0.053*	
C14	−0.0353 (6)	0.5529 (5)	0.7377 (5)	0.0457 (12)	
H15	−0.1196	0.5684	0.8078	0.055*	
Cd1	0.17488 (4)	0.24849 (4)	0.84349 (3)	0.03544 (14)	
N1	0.3893 (4)	0.0667 (4)	0.8096 (3)	0.0354 (9)	
N2	0.3429 (4)	0.2738 (4)	0.6467 (3)	0.0326 (8)	
N3	0.1732 (4)	0.4647 (4)	0.6135 (3)	0.0358 (9)	
N4	0.0804 (4)	0.4489 (4)	0.7295 (3)	0.0412 (9)	
N5	0.3227 (5)	0.6794 (5)	0.2041 (4)	0.0526 (11)	
N6	−0.0540 (5)	0.2346 (5)	1.0734 (4)	0.0466 (11)	
N7	−0.0228 (5)	0.6358 (5)	0.6366 (4)	0.0532 (11)	
O1	0.0636 (4)	0.1389 (4)	1.0457 (3)	0.0549 (9)	
O2	−0.0577 (4)	0.3376 (4)	1.0003 (3)	0.0519 (9)	
O3	−0.1586 (5)	0.2266 (5)	1.1677 (4)	0.0750 (13)	
O4	0.2077 (5)	0.7114 (5)	0.2931 (4)	0.0688 (12)	
O5	0.4194 (5)	0.5758 (6)	0.2111 (4)	0.0840 (15)	
O6	0.3399 (6)	0.7527 (6)	0.1125 (4)	0.0923 (16)	
O7	0.0187 (4)	0.1051 (4)	0.7810 (3)	0.0493 (9)	
H6	−0.0719	0.1640	0.7649	0.074*	
H7	0.0163	0.0305	0.8149	0.074*	
O8	0.2817 (4)	0.4079 (4)	0.9389 (3)	0.0524 (9)	
H4	0.2302	0.4922	0.9605	0.079*	
H5	0.3553	0.4366	0.9016	0.079*	

### Atomic displacement parameters (Å^2^)

**Table:**

	*U*^11^	*U*^22^	*U*^33^	*U*^12^	*U*^13^	*U*^23^
C1	0.048 (3)	0.039 (3)	0.041 (3)	0.007 (2)	−0.009 (2)	0.005 (2)
C2	0.067 (4)	0.044 (3)	0.051 (3)	0.016 (3)	−0.019 (3)	0.009 (2)
C3	0.049 (3)	0.046 (3)	0.058 (3)	0.016 (3)	−0.016 (3)	−0.002 (3)
C4	0.033 (2)	0.037 (3)	0.047 (3)	0.005 (2)	−0.010 (2)	−0.009 (2)
C5	0.034 (2)	0.029 (2)	0.035 (2)	0.0005 (19)	−0.0067 (19)	−0.0042 (18)
C6	0.028 (2)	0.032 (2)	0.034 (2)	0.0007 (19)	−0.0043 (18)	−0.0033 (18)
C7	0.033 (2)	0.037 (3)	0.034 (2)	−0.006 (2)	0.0010 (19)	−0.0066 (19)
C8	0.034 (3)	0.050 (3)	0.043 (3)	0.003 (2)	0.007 (2)	−0.016 (2)
C9	0.030 (3)	0.054 (3)	0.058 (3)	0.009 (2)	−0.001 (2)	−0.019 (3)
C10	0.044 (3)	0.049 (3)	0.026 (2)	−0.006 (2)	0.006 (2)	−0.002 (2)
C11	0.048 (3)	0.040 (3)	0.029 (2)	−0.008 (2)	−0.005 (2)	0.0066 (19)
C12	0.035 (2)	0.028 (2)	0.033 (2)	−0.0020 (19)	−0.0048 (19)	−0.0012 (18)
C13	0.051 (3)	0.034 (3)	0.046 (3)	0.003 (2)	−0.017 (2)	0.011 (2)
C14	0.044 (3)	0.037 (3)	0.048 (3)	0.011 (2)	−0.007 (2)	0.004 (2)
Cd1	0.0326 (2)	0.0334 (2)	0.0298 (2)	0.00505 (14)	0.00361 (13)	0.00419 (13)
N1	0.0320 (19)	0.037 (2)	0.032 (2)	0.0049 (17)	−0.0044 (16)	0.0055 (16)
N2	0.0308 (19)	0.031 (2)	0.030 (2)	0.0008 (16)	−0.0023 (16)	0.0008 (15)
N3	0.036 (2)	0.031 (2)	0.037 (2)	0.0000 (17)	−0.0067 (17)	0.0050 (16)
N4	0.042 (2)	0.036 (2)	0.035 (2)	0.0051 (18)	0.0005 (17)	0.0052 (16)
N5	0.052 (3)	0.055 (3)	0.053 (3)	−0.013 (2)	−0.017 (2)	0.017 (2)
N6	0.047 (2)	0.046 (3)	0.036 (2)	−0.014 (2)	0.0107 (19)	−0.0077 (19)
N7	0.051 (3)	0.044 (3)	0.060 (3)	0.011 (2)	−0.016 (2)	0.006 (2)
O1	0.059 (2)	0.046 (2)	0.047 (2)	0.0010 (19)	0.0014 (18)	0.0038 (16)
O2	0.050 (2)	0.043 (2)	0.049 (2)	0.0030 (17)	0.0043 (17)	0.0016 (17)
O3	0.068 (3)	0.078 (3)	0.052 (3)	−0.016 (2)	0.027 (2)	0.000 (2)
O4	0.058 (2)	0.088 (3)	0.055 (2)	−0.015 (2)	−0.006 (2)	0.010 (2)
O5	0.061 (3)	0.088 (4)	0.091 (3)	0.010 (3)	−0.014 (2)	0.034 (3)
O6	0.095 (4)	0.106 (4)	0.063 (3)	0.003 (3)	−0.012 (3)	0.041 (3)
O7	0.0464 (19)	0.041 (2)	0.057 (2)	−0.0010 (16)	−0.0103 (17)	0.0037 (16)
O8	0.048 (2)	0.045 (2)	0.059 (2)	0.0000 (17)	−0.0091 (18)	−0.0049 (17)

### Geometric parameters (Å, °)

**Table:**

C1—N1	1.316 (6)	C13—N7	1.309 (7)
C1—C2	1.396 (7)	C13—N3	1.347 (6)
C1—H1	0.9300	C13—H13	0.9300
C2—C3	1.331 (7)	C14—N4	1.313 (6)
C2—H2	0.9300	C14—N7	1.352 (7)
C3—C4	1.401 (7)	C14—H15	0.9300
C3—H3	0.9300	Cd1—O7	2.301 (4)
C4—C5	1.401 (6)	Cd1—O8	2.303 (4)
C4—C9	1.429 (7)	Cd1—N2	2.340 (3)
C5—N1	1.362 (5)	Cd1—N1	2.350 (4)
C5—C6	1.424 (6)	Cd1—O2	2.400 (3)
C6—N2	1.353 (5)	Cd1—N4	2.445 (4)
C6—C7	1.407 (6)	Cd1—O1	2.475 (4)
C7—C10	1.392 (7)	N3—N4	1.361 (5)
C7—C8	1.436 (6)	N5—O6	1.212 (6)
C8—C9	1.337 (8)	N5—O5	1.231 (6)
C8—H8	0.9300	N5—O4	1.235 (6)
C9—H9	0.9300	N6—O3	1.214 (5)
C10—C11	1.357 (7)	N6—O2	1.251 (6)
C10—H10	0.9300	N6—O1	1.265 (5)
C11—C12	1.395 (6)	O7—H6	0.9858
C11—H11	0.9300	O7—H7	0.7785
C12—N2	1.309 (6)	O8—H4	0.8595
C12—N3	1.411 (6)	O8—H5	0.7473
			
N1—C1—C2	123.4 (5)	O7—Cd1—N1	94.70 (13)
N1—C1—H1	118.3	O8—Cd1—N1	94.71 (13)
C2—C1—H1	118.3	N2—Cd1—N1	70.66 (12)
C3—C2—C1	119.1 (5)	O7—Cd1—O2	86.19 (13)
C3—C2—H2	120.4	O8—Cd1—O2	81.33 (13)
C1—C2—H2	120.4	N2—Cd1—O2	148.68 (12)
C2—C3—C4	120.5 (5)	N1—Cd1—O2	140.60 (13)
C2—C3—H3	119.7	O7—Cd1—N4	87.39 (14)
C4—C3—H3	119.7	O8—Cd1—N4	91.19 (14)
C5—C4—C3	117.0 (5)	N2—Cd1—N4	66.93 (12)
C5—C4—C9	118.8 (4)	N1—Cd1—N4	137.57 (12)
C3—C4—C9	124.2 (4)	O2—Cd1—N4	81.82 (12)
N1—C5—C4	122.4 (4)	O7—Cd1—O1	84.17 (13)
N1—C5—C6	118.8 (4)	O8—Cd1—O1	87.88 (13)
C4—C5—C6	118.9 (4)	N2—Cd1—O1	158.95 (13)
N2—C6—C7	120.6 (4)	N1—Cd1—O1	88.53 (12)
N2—C6—C5	117.8 (4)	O2—Cd1—O1	52.30 (12)
C7—C6—C5	121.6 (4)	N4—Cd1—O1	133.72 (12)
C10—C7—C6	117.4 (4)	C1—N1—C5	117.6 (4)
C10—C7—C8	125.0 (4)	C1—N1—Cd1	126.6 (3)
C6—C7—C8	117.5 (4)	C5—N1—Cd1	115.7 (3)
C9—C8—C7	121.1 (4)	C12—N2—C6	119.6 (4)
C9—C8—H8	119.5	C12—N2—Cd1	123.5 (3)
C7—C8—H8	119.5	C6—N2—Cd1	116.9 (3)
C8—C9—C4	122.1 (4)	C13—N3—N4	108.9 (4)
C8—C9—H9	118.9	C13—N3—C12	130.9 (4)
C4—C9—H9	118.9	N4—N3—C12	120.2 (4)
C11—C10—C7	121.6 (4)	C14—N4—N3	102.6 (4)
C11—C10—H10	119.2	C14—N4—Cd1	142.7 (3)
C7—C10—H10	119.2	N3—N4—Cd1	114.7 (3)
C10—C11—C12	116.8 (4)	O6—N5—O5	121.3 (5)
C10—C11—H11	121.6	O6—N5—O4	119.3 (5)
C12—C11—H11	121.6	O5—N5—O4	119.4 (5)
N2—C12—C11	123.8 (4)	O3—N6—O2	121.5 (5)
N2—C12—N3	114.6 (4)	O3—N6—O1	121.1 (5)
C11—C12—N3	121.6 (4)	O2—N6—O1	117.4 (4)
N7—C13—N3	110.5 (4)	C13—N7—C14	103.0 (4)
N7—C13—H13	124.8	N6—O1—Cd1	93.0 (3)
N3—C13—H13	124.8	N6—O2—Cd1	97.0 (3)
N4—C14—N7	115.0 (5)	Cd1—O7—H6	111.8
N4—C14—H15	122.5	Cd1—O7—H7	110.1
N7—C14—H15	122.5	H6—O7—H7	125.4
O7—Cd1—O8	167.51 (12)	Cd1—O8—H4	117.9
O7—Cd1—N2	94.13 (12)	Cd1—O8—H5	116.9
O8—Cd1—N2	96.72 (13)	H4—O8—H5	95.7
			
N1—C1—C2—C3	0.9 (9)	O8—Cd1—N2—C12	−91.5 (4)
C1—C2—C3—C4	−2.3 (9)	N1—Cd1—N2—C12	175.9 (4)
C2—C3—C4—C5	2.1 (8)	O2—Cd1—N2—C12	−7.1 (5)
C2—C3—C4—C9	−175.3 (5)	N4—Cd1—N2—C12	−3.1 (3)
C3—C4—C5—N1	−0.4 (7)	O1—Cd1—N2—C12	166.8 (4)
C9—C4—C5—N1	177.1 (4)	O7—Cd1—N2—C6	−95.1 (3)
C3—C4—C5—C6	−178.9 (5)	O8—Cd1—N2—C6	91.0 (3)
C9—C4—C5—C6	−1.4 (7)	N1—Cd1—N2—C6	−1.6 (3)
N1—C5—C6—N2	3.6 (6)	O2—Cd1—N2—C6	175.4 (3)
C4—C5—C6—N2	−177.9 (4)	N4—Cd1—N2—C6	179.5 (3)
N1—C5—C6—C7	−175.7 (4)	O1—Cd1—N2—C6	−10.6 (6)
C4—C5—C6—C7	2.8 (7)	N7—C13—N3—N4	−0.7 (6)
N2—C6—C7—C10	−3.0 (7)	N7—C13—N3—C12	179.5 (5)
C5—C6—C7—C10	176.2 (5)	N2—C12—N3—C13	177.4 (5)
N2—C6—C7—C8	178.6 (4)	C11—C12—N3—C13	−1.7 (8)
C5—C6—C7—C8	−2.2 (7)	N2—C12—N3—N4	−2.3 (6)
C10—C7—C8—C9	−178.3 (5)	C11—C12—N3—N4	178.5 (4)
C6—C7—C8—C9	0.0 (7)	N7—C14—N4—N3	−1.0 (6)
C7—C8—C9—C4	1.5 (8)	N7—C14—N4—Cd1	−179.5 (4)
C5—C4—C9—C8	−0.8 (8)	C13—N3—N4—C14	1.0 (5)
C3—C4—C9—C8	176.5 (5)	C12—N3—N4—C14	−179.2 (4)
C6—C7—C10—C11	2.8 (7)	C13—N3—N4—Cd1	180.0 (3)
C8—C7—C10—C11	−178.9 (5)	C12—N3—N4—Cd1	−0.2 (5)
C7—C10—C11—C12	−1.0 (7)	O7—Cd1—N4—C14	84.3 (6)
C10—C11—C12—N2	−0.8 (7)	O8—Cd1—N4—C14	−83.3 (6)
C10—C11—C12—N3	178.3 (4)	N2—Cd1—N4—C14	179.9 (6)
C2—C1—N1—C5	0.7 (8)	N1—Cd1—N4—C14	178.4 (5)
C2—C1—N1—Cd1	−176.5 (4)	O2—Cd1—N4—C14	−2.2 (6)
C4—C5—N1—C1	−0.9 (7)	O1—Cd1—N4—C14	4.9 (7)
C6—C5—N1—C1	177.6 (4)	O7—Cd1—N4—N3	−94.1 (3)
C4—C5—N1—Cd1	176.6 (3)	O8—Cd1—N4—N3	98.3 (3)
C6—C5—N1—Cd1	−5.0 (5)	N2—Cd1—N4—N3	1.5 (3)
O7—Cd1—N1—C1	−86.6 (4)	N1—Cd1—N4—N3	0.0 (4)
O8—Cd1—N1—C1	85.1 (4)	O2—Cd1—N4—N3	179.4 (3)
N2—Cd1—N1—C1	−179.4 (4)	O1—Cd1—N4—N3	−173.5 (3)
O2—Cd1—N1—C1	3.1 (5)	N3—C13—N7—C14	0.1 (6)
N4—Cd1—N1—C1	−177.9 (4)	N4—C14—N7—C13	0.6 (7)
O1—Cd1—N1—C1	−2.6 (4)	O3—N6—O1—Cd1	175.4 (4)
O7—Cd1—N1—C5	96.1 (3)	O2—N6—O1—Cd1	−4.9 (4)
O8—Cd1—N1—C5	−92.1 (3)	O7—Cd1—O1—N6	−86.8 (3)
N2—Cd1—N1—C5	3.4 (3)	O8—Cd1—O1—N6	83.5 (3)
O2—Cd1—N1—C5	−174.1 (3)	N2—Cd1—O1—N6	−173.2 (3)
N4—Cd1—N1—C5	4.9 (4)	N1—Cd1—O1—N6	178.3 (3)
O1—Cd1—N1—C5	−179.8 (3)	O2—Cd1—O1—N6	2.8 (3)
C11—C12—N2—C6	0.5 (7)	N4—Cd1—O1—N6	−6.1 (4)
N3—C12—N2—C6	−178.6 (4)	O3—N6—O2—Cd1	−175.2 (4)
C11—C12—N2—Cd1	−176.8 (3)	O1—N6—O2—Cd1	5.1 (5)
N3—C12—N2—Cd1	4.0 (5)	O7—Cd1—O2—N6	82.7 (3)
C7—C6—N2—C12	1.4 (6)	O8—Cd1—O2—N6	−96.9 (3)
C5—C6—N2—C12	−177.9 (4)	N2—Cd1—O2—N6	174.4 (3)
C7—C6—N2—Cd1	179.0 (3)	N1—Cd1—O2—N6	−10.1 (4)
C5—C6—N2—Cd1	−0.3 (5)	N4—Cd1—O2—N6	170.6 (3)
O7—Cd1—N2—C12	82.3 (4)	O1—Cd1—O2—N6	−2.9 (3)

### Hydrogen-bond geometry (Å, °)

**Table:**

*D*—H···*A*	*D*—H	H···*A*	*D*···*A*	*D*—H···*A*
O8—H5···O5^i^	0.75	2.05	2.752 (6)	156
O8—H4···O2^ii^	0.86	2.03	2.865 (5)	163
O7—H7···O3^iii^	0.78	2.59	3.269 (6)	147
O7—H7···O1^iii^	0.78	2.22	2.966 (5)	160
O7—H6···N5^iv^	0.99	2.47	3.420 (6)	162
O7—H6···O6^iv^	0.99	2.45	3.227 (6)	135
O7—H6···O4^iv^	0.99	1.83	2.802 (6)	167

Symmetry codes: (i) −*x*+1, −*y*+1, −*z*+1; (ii) −*x*, −*y*+1, −*z*+2; (iii) −*x*, −*y*, −*z*+2; (iv) −*x*, −*y*+1, −*z*+1.
